# 高龄肺鳞癌患者的临床病理特征

**DOI:** 10.3779/j.issn.1009-3419.2016.10.07

**Published:** 2016-10-20

**Authors:** 洁 陈, 学志 郝, 芳 程, 童童 张, 镨元 邢, 峻岭 李

**Affiliations:** 1 100122 北京，北京市朝阳区三环肿瘤医院 Beijing Chaoyang Sanhuan Cancer Hospital, Beijing 100122, China; 2 100021 北京，国家癌症中心/中国医学科学院北京协和医学院肿瘤医院 National Cancer Center/Cancer Hospital, Chinese Academy of Medical Sciences and Peking Union Medical College, Beijing 100021, China

**Keywords:** 肺肿瘤, 鳞癌, 病理特征, Lung neoplasms, Squamous cell carcinoma, Pathological characteristics

## Abstract

**背景与目的:**

随着人口老龄化及烟草的流行，老年肺癌患者的发病率呈上升趋势。但在各种临床实验中老年（≥70岁）患者不入组或很少入组，使得老年肺鳞癌患者的临床研究证据不足。本研究以80岁患者为界，观察分析高龄肺鳞癌患者的临床特征、治疗方法及影响治疗的因素并探讨高龄肺鳞癌患者治疗的选择。

**方法:**

回顾性分析38例老年高龄肺鳞癌患者的临床特征，总结高龄肺鳞癌患者在诊断及临床分期明确的情况下选择治疗方式。

**结果:**

老年高龄鳞癌患者在身体状况可以耐受的情况下，可以根据患者的诊断及临床分期选择手术、放疗及化疗。

**结论:**

老年高龄患者由于其生存期较短，能够接受的有效及完整的治疗较老年（70岁-80岁）患者要少，≥80岁的肺鳞癌患者在其身体状况允许及不影响生活质量的情况下，根据其病情分期应选择最佳的治疗。

## Abstract (en)

**Background and objective:**

As the prevalence of tobacco and the aging of the population, the incidence of lung cancer in the elderly rises. However, few elderly patients (older than 70 years old) with lung squamous cell carcinoma were involved into the clinical trials, which offered insufficient clinical evidence for these patients. Lung squamous cell carcinoma patients older than 80 years old were included in our study to analyze the clinical characteristics, treatment and prognostic factors, and to explore the optimal treatment choices for these patients.

**Methods:**

We retrospectively analyzed the clinical features of 38 elderly patients with lung squamous carcinoma and summarized the treatment under the clear diagnosis and clinical staging.

**Results:**

Elderly patients with squamous cell carcinoma can choose surgery, radiotherapy and chemotherapy based on diagnosis and clinical staging when their physical condition is permitted.

**Conclusion:**

Because of the short life expectancy of patients more than 80 years old, fewer of them could receive completed and effective treatment, comparing with patients between 70 and 80 years old.

目前，肺癌是全世界范围内肿瘤死亡的首要原因^[1]^，在我国肺癌为癌症发病率和死亡率上升最快的肿瘤^[2]^。吸烟^[3]^是公认的肺癌高危因素，他与多数肺癌相关死亡有关，由于烟草的流行、医疗水平的提高及人口老龄化，肺癌的发病率及死亡率持续上升，因此老年肺癌患者数目不断增加。非小细胞肺癌（non-small cell lung cancer, NSCLC）占肺癌的85%以上，其中鳞状细胞癌（squamous cell lung cancer, SCC）约占NSCLC的30%^[4]^，预后较差；而超过2/3的患者年龄大于65岁^[5]^。因此，肺癌可以被视为常见的老年病。目前，肺鳞癌的主要治疗方式是手术、放疗、化疗及免疫治疗，但是老年患者由于身体状况及治疗耐受性较差，往往不能接受有效的完整治疗^[6]^，从而使老年患者的肺癌相关死亡风险上升^[7]^。本研究通过分析高龄肺鳞癌患者的临床病理特征及治疗方式，以期为今后的临床治疗提供依据。

## 1 材料与方法

回顾性分析2011年1月-2014年12月在中国医学科学院肿瘤医院经病理或细胞学确诊的38例高龄（年龄≥80岁）肺鳞癌患者，其中男性32例，女性6例，年龄范围为80岁-92岁，平均年龄为82.2岁；其中吸烟患者32例，非吸烟患者6例；按照国际肺癌研究协会（International Association for the Study of Lung Cancer, IASLC）2009年颁布的第七版肺癌肿瘤-淋巴结-转移（tumor-node-metastasis, TNM）分期标准进行临床分期，其中Ⅰ期-Ⅲa期32例，Ⅲb期-Ⅳ期6例。

## 2 结果

### 2.1 临床病理特征

38例患者中美国东部肿瘤协作组（Eastern Cooperative Oncology Group, ECOG）评分为0分13例（34.2%），1分22例（57.9%），2分3例（7.9%）。病理分级：低分化9例（23.7%），中分化9例（23.7%），高分化2例（5.3%），分化不明确18例（47.4%）。合并基础疾病的共29例（76.3%），其中22例（57.9%）合并心血管疾病，6例（15.8%）合并糖尿病，21例（55.3%）合并其他基础疾病。

### 2.2 治疗情况

首诊为Ⅰ期-Ⅲa期患者共32例（84.3%），其中14例（43.8%）接受手术治疗，18例（56.2%）接受局部放疗的患者；Ⅲb期患者3例，1例（33.3%）仅接受了全身化疗，2例（66.7%）仅接受了局部放疗；Ⅳ期患者1例，仅接受了局部姑息性放疗。

1例首诊为Ⅲb期的患者仅接受了全身化疗，一线方案为吉西他滨联合顺铂，疗效评价病情进展（progressive disease, PD），耐受性良好，二线治疗方案为紫杉醇脂质体联合异环磷酰胺，疗效评价为PD，耐受性良好。本研究中2例首诊为Ⅰ期-Ⅲa期的患者在疾病复发或出现远处转移时均选择了全身化疗，1例患者一线方案为多西他赛联合泰欣生，后多西他赛单药维持治疗，疗效评价为疾病稳定（stable disease, SD），无进展生存期（progression-free survival, PFS）达到了11.7个月，耐受性良好；另1例患者一线方案选择了白蛋白结合型紫杉醇单药周疗，疗效评价SD，PFS为7.0个月，耐受性良好（表 1）。

**1:** 患者基线特征 Baseline demographic and disease characteristics

Characteristics	No. of patients	Percent (%)
Gender		
Male	32	84.2
Female	6	15.8
Cigarette smoking		
Yes	32	84.2
No	6	15.8
ECOG score		
0	13	34.2
1	22	57.9
2	3	7.9
TNM stage		
Ⅰ	16	42.1
Ⅱ	8	21.1
Ⅲa	8	21.1
Ⅲb	3	7.9
Ⅳ	3	7.9
Pathological grade		
Low	9	23.7
Moderate	9	23.7
High	2	5.3
Comorbidities		
Hypertension and cardiac disease	22	57.9
Diabetes mellitus	6	15.8
Others	21	55.3
ECOG: Eastern Cooperative Oncology Group; TNM: tumor-node-metastasis.		

## 3 讨论

肺癌是全世界范围内肿瘤死亡的首要原因，吸烟是肺癌的主要危险因素，它与多数肺癌相关性死亡有关，烟草中包含多种致癌化学物质^[8]^，随着每天吸烟支数以及吸烟年数的增加，患肺癌的危险也相应增加，尤其是男性患者。本研究中，约50%的高龄肺鳞癌患者有长期吸烟史，烟龄最长患者的吸烟指数可达94.5包年，男性吸烟者要明显多于女性，这也证实了吸烟与肺癌发生相关，尤其是与男性肺癌患者关系更加密切。另外，有研究^[9]^报道了非吸烟者因“二手烟”而发展成肺癌的危险比（HR=1.05），这也正是女性肺癌发生率逐年升高的重要原因。在本研究中，有一半的女性患者为非吸烟者，但均有被动吸烟史，这进一步说明二手烟是非吸烟女性肺癌发生的重要危险因素。

晚期NSCLC患者吸烟指数（包年）越高，生存期越短^[10]^。Ferketich等^[11]^发现：Ⅰ期-Ⅲa期的NSCLC非吸烟者较吸烟者的总生存（overall survival, OS）要更长，而Ⅳ期患者从不吸烟因素中的生存获益随着年龄的增长而降低，当患者年龄＞85岁时，吸烟对于OS的影响无统计学差异^[11]^。本研究由于样本量较少，随访时间较短，无法统计吸烟对高龄肺鳞癌患者的生存及预后是否有影响，仍需进一步的研究数据加以证实。

由于大多临床研究的入组标准都排除了高龄患者，因此，目前有关高龄NSCLC患者的临床数据有限。高龄（≥80岁）患者生存预期较短、治疗风险较大、治疗获益有限，因此，这类患者在晚期时往往倾向于安全性较好的姑息治疗，而放弃全身治疗。ELVIS^[12]^研究是第一项针对老年的Ⅲ期随机对照研究，他首次证明了化疗较安慰剂能够显著延长老年患者的OS（28周*vs*21周，*P*=0.03）。IFCT-0501^[13]^研究首次证明与单药方案相比，含铂双药方案能够延长PS评分0分-2分老年患者的OS（10.3周*vs*6.2周，*P*＜0.000, 1）。2012年的一项荟萃分析结果^[14]^显示：对于老年患者，含铂双药方案的疗效优于单药方案，可降低约16%的死亡风险，至疾病进展时间（time to progression, TTP）、客观缓解率（objective response rate, ORR）、1年生存率均明显优于单药。本研究中大部分患者的ECOG评分均为0分-1分，但仅3例患者一线治疗选择全身化疗，其中1例患者接受含铂双药方案，另2例患者选择紫杉类药物，3例患者治疗耐受性均良好，因此全身化疗仍可以作为高龄肺鳞癌患者的一个治疗选择。但由于样本量较少，无法进行统计分析，从而证实联合化疗方案在老年患者中的优势，仍需大样本的前瞻性研究加以证实。

目前，国际上对老年患者的治疗并没有提出标准的治疗方案。早期老年NSCLC患者是否进行手术仍存在争议。放疗因安全性较高，可以作为手术之外的一个局部治疗选择，同时可以达到和手术类似的效果。美国国立综合癌症网络（National Comprehensive Cancer Network, NCCN）指南推荐：放疗是Ⅰ期-Ⅱ期老年NSCLC患者的首选治疗。目前，高效低毒的第三代化疗药物已成为老年晚期NSCLC患者的标准治疗，而其联合铂类的化疗方案也是一个安全有效的治疗选择^[15]^。年龄和合并基础疾病可能是影响临床医生、患者及家属选择治疗的最大因素。已往的肺癌研究^[16-18]^显示，在治疗的选择上，年龄比合并疾病更加重要。本研究中3例接受全身化疗的高龄肺鳞癌患者均合并基础疾病，但化疗耐受性良好。因此，合并基础疾病不能作为高龄肺鳞癌患者全身化疗的禁忌，高龄肺鳞癌患者的治疗需要每位临床医生根据具体情况制定相应的治疗方案。
